# Sex Specific Effects of a High Fat Diet on Metabolism, Cognition, and Pathology in the Tg-SwDI Mouse Model of Alzheimer’s Disease

**DOI:** 10.21203/rs.3.rs-7686971/v1

**Published:** 2025-10-10

**Authors:** Shelby Sabourin, Christina Thrasher, Rachel Smith, Kasey Belanger-Mayer, Bryce Thibodeau, Richard Kelly, Riane Richard, Abigail Salinero, Charly Abi-Ghanem, Molly Batchelder, Emily Groom, Sally Temple, Kevin Pumiglia, Kristen Zuloaga

**Affiliations:** Albany Medical College; Albany Medical College; Albany Medical College; Albany Medical College; Albany Medical College; Albany Medical College; Albany Medical College; Albany Medical College; Albany Medical College; Albany Medical College; Albany Medical College; Neural Stem Cell Institute; Albany Medical College; Albany Medical College

**Keywords:** Alzheimer’s disease, metabolism, neuroinflammation, sex, cerebral amyloid angiopathy, vascular, dementia, VCID, obesity, prediabetes

## Abstract

**Background:**

Alzheimer’s disease (AD) is the leading cause of dementia in the US, with over 80% of affected individuals experiencing comorbid metabolic disease. Along with age and sex, metabolic syndrome and prediabetes are known risk factors for developing dementia and AD, highlighting the complex nature of the disease. How these risk factors affect cerebral amyloid angiopathy (CAA) is less well studied. As such, we examined the effect of diet-induced metabolic syndrome and sex on cognition, neuroinflammation, and pathology in the Tg-SwDI mouse model of AD and CAA.

**Methods:**

Male and female Tg-SwDI and WT mice were fed a low fat (LFD; 10% fat) or high fat (HFD; 60% fat) diet from 3 to 10 months of age. Metabolic, cognitive, and neuropathology outcomes were assessed.

**Results:**

All HFD-fed mice gained weight and exhibited impaired glucose tolerance. Metabolic disturbances were most severe in AD females receiving HFD. In both males and females, HFD-fed AD mice showed increased anxiety-like behavior, decreased locomotor activity, and impaired episodic memory in the open field and novel object recognition tests, respectively. HFD-fed AD females specifically exhibited spatial memory deficits in the Barnes maze. Hippocampal microgliosis, activated microglia, and astrogliosis were more severe in AD mice, but this effect was blunted by HFD in females in the cornu ammonis 1. HFD-fed AD females had greater amyloid plaques and CAA in the thalamus compared to LFD-fed AD controls. All metrics of neuroinflammation significantly correlated with CAA pathology in the thalamus.

**Conclusion:**

AD females experienced greater metabolic, cognitive, and pathologic effects in response to a HFD compared to AD males and WT controls. These observations provide a better understanding of how metabolic disease may differentially affect the development of dementia in men and women.

## BACKGROUND

Alzheimer’s disease (AD) is the most common cause of dementia, with over 55 million people affected worldwide(1). Risk factors such as age, sex, cardiovascular disease, and genetics highlight the complex nature of this disease(2). Currently, over 80% of individuals with AD have comorbid metabolic disease(3–5). Further, there is ample and growing evidence that prediabetes and metabolic syndrome increase the risk of developing dementia(2, 5–7). Conservative estimates suggest the prevalence of these metabolic conditions is around 10% globally(8, 9). Characterized by insulin resistance, hyperglycemia, hyperlipidemia, hypertension, obesity, or impaired glucose tolerance, these metabolic conditions greatly increase the risk for developing diabetes and cardiovascular disease(9). Impaired metabolism can cause oxidative stress and persistent inflammation, which are pathological hallmarks shared with AD(7).

In both humans and animal models, metabolic disease has been associated with cognitive impairment(2). Following long-term access to a high-fat diet (HFD), mice exhibit weight gain, insulin resistance, glucose tolerance, and ultimately cognitive decline(10, 11). Our laboratory and others have shown that HFD-induced metabolic disease causes cognitive deficits, reduces cerebral blood flow, and increases inflammation in wild type (WT) mice and that these deficits can be improved by reversing dietary fat or administering healthy plasma(11–13). We have also shown that disease pathology and behavioral deficits are exacerbated by HFD in mouse models of dementia in a sex-specific manner, such that these effects are worse in females(14–16).

Due to the complexity of this disease, there is a large, ongoing effort to characterize AD models to better understand pathophysiology, as well as identify models suitable for investigating potential therapeutics. Often those with AD have both parenchymal amyloid beta plaques and amyloid beta accumulation within cerebral vessels, known as cerebral amyloid angiopathy (CAA)(17). The Tg-SwDl mice are a transgenic mouse model with both traditional AD pathology of parenchymal amyloid beta plaques and CAA pathology(18). We hypothesized that the Tg-SwDl mouse model will have worse cognitive impairment than wild type control mice and that metabolic disease will exacerbate these impairments more so in females than males. This study is the first to characterize sex differences in the impact of a high fat diet on metabolism, cognition, and pathology in the Tg-SwDl transgenic model of AD with CAA. This study was ultimately designed to further characterize this AD mouse model and highlight sex and metabolic health as key biological variables.

## METHODS

### Animals and experimental design

**Figure 1.**
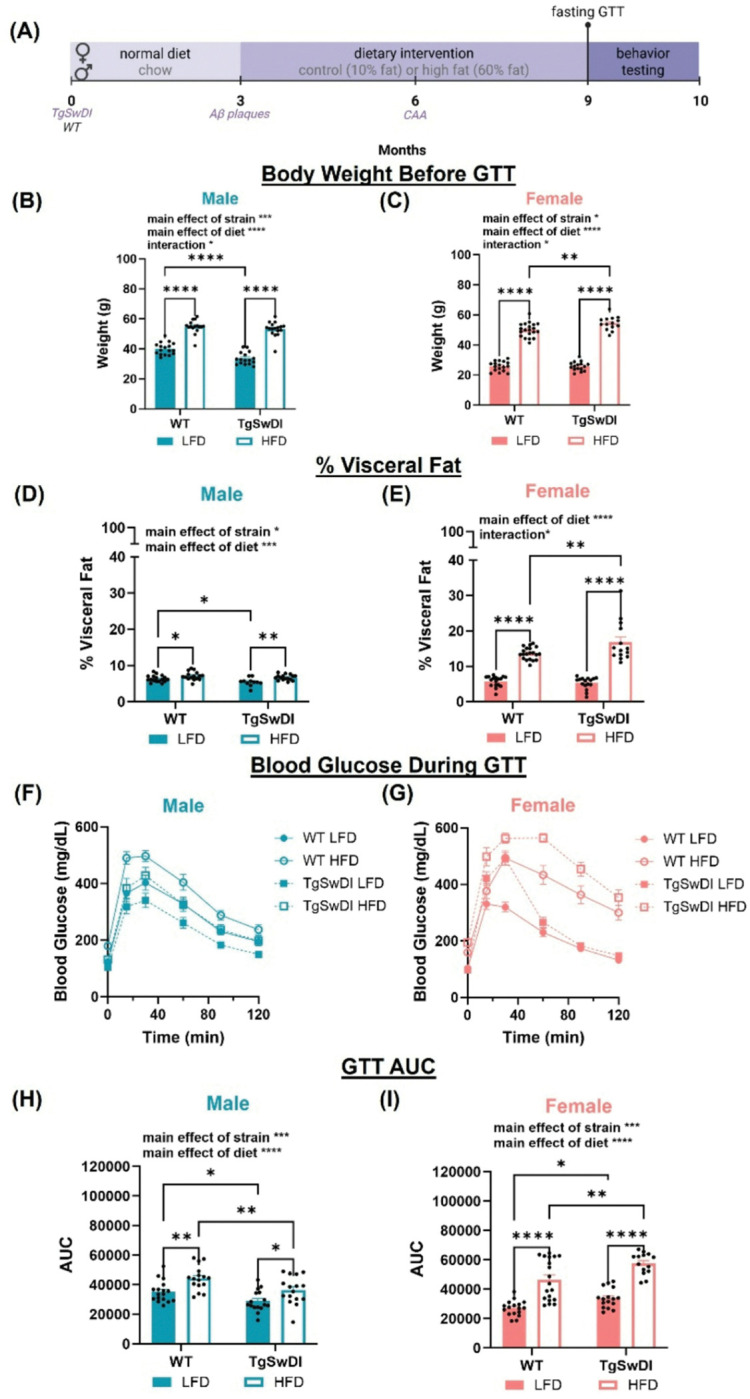
Diet affects weight, visceral adiposity, and glucose tolerance in WT and Tg-SwDI males and females. (A) The experimental timeline is shown. (B, C) Body weight prior to the GTT and following 6 months of dietary intervention was used to assess weight gain. (D, E) Wet weight (g) of visceral fat was assessed at the end of the experiment and analyzed relative to percent of total body weight at that time. (F-I) GTT was performed to assess metabolism and diabetic state. (F, G) After overnight fasting (t=0), blood glucose levels were measured, and mice were injected with a glucose challenge during which blood glucose was measured at 15, 30, 60, 90, and 120 minutes. (H, I) Area under the curve (AUC) from GTT was assessed as a measure of responsiveness to the glucose challenge. Higher AUC is indicative of worse metabolic disease. * p<0.05; ** p<0.01; **** p<0.0001. Two-way ANOVA (n=11–20/group).

This study was conducted in accordance with the National Institutes of Health Guidelines for the Care and Use of Laboratory Animals, and protocols were approved by the Institutional Animal Care and Use Committee at Albany Medical College (Albany, NY, USA). In the animal facility, temperature and humidity were set at 72°F, 30–70% humidity, with a 12-h light/dark cycle (7 a.m. on/7 p.m. off). Mice were fed a standard chow diet (Purina Lab Diet 5P76) until three months of age. They were group housed in Allentown cages. Environmental enrichment (Nestlets and Shepherd Shacks) was provided, and mice were group housed at all times. Male and female wild type (WT; N = 73; C57BL/6J; #000664) and Tg-SwDl transgenic mice (Tg-SwDl; N = 66, C57BL/6-Tg(Thy1-APPSwDutIowa)BWevn/Mmjax; #034843; MMRRC_034843-JAX) were purchased from Jackson Laboratories (Bar Harbor, ME). Tg-SwDl mice were then used to maintain a colony at Albany Medical Center’s Animal Resource Facility. These transgenic mice express the human APP gene containing the Swedish K670N/M671L, Dutch E693Q, and Iowa D694N mutations regulated by the *Thy1* promoter(18). Starting at approximately three months of age, the mice develop Aβ deposition in the parenchyma and learning and memory deficits(18–20). At around six months of age amyloid begins to accumulate in cerebral vessels. A timeline of the experiment is shown in Fig. 1A. At 3 months of age, mice were placed on either a HFD (60% fat, 5.24 kcal/g; D12492, Research Diets, New Brunswick, NJ) or a low-fat control diet (LFD) (10% fat, 3.82 kcal/g; D12450J, Research Diets) until the end of the study. Mice were weighed every 4 weeks throughout the diet. At 9 months, mice underwent a glucose tolerance test (GTT), followed by a 2-week rest period and behavioral testing. After testing, animals were euthanized, and tissue was collected at 10 months of age. Experiments were conducted in cohorts of up to 25 mice with a total of 139 mice. In total, 7 mice were excluded due to premature death or the presence of other major health exclusions (hydrocephaly, large fighting wounds, tumors). The remaining 132 brains were post-fixed for immunohistochemistry. Blinding to diet and sex was not possible during in vivo experiments due to mouse appearance. During analysis, experimenters were blinded to sex, diet, and genotype.

### Glucose tolerance test

As previously described, mice were given a GTT to assess metabolic status at 9 months of age(13, 16, 21). The mice were fasted overnight, and their fasting blood glucose levels (t = 0) were measured from their tail vein the next morning using a glucometer (Verio IQ, OneTouch, Sunnyvale, CA, USA). Following an intraperitoneal injection of 2 g/kg of glucose, blood glucose levels were measured at 15, 30, 60, 90, and 120 minutes post-injection to assess metabolic response to a glucose challenge.

### Behavioral testing

Following a 2-week recovery period, mice underwent testing for exploratory activity and anxiety-like behavior in the open field (day 1), episodic-like memory in the novel object recognition test (NORT; day 2), and spatial learning and memory in the Barnes maze (days 8–13). For each assessment, mice were acclimated to the light in the procedure room for 1 hour prior to testing. Between each animal, 70% ethanol was used to clean the apparatus to remove olfactory cues. During each test, videos were recorded independently analyzed using automated retracking software (ANY-maze 7.0, Stoelting, Wood Dale, IL).

### Open field

Mice were placed in the testing apparatus (495 × 495 mm box) for 10 minutes in total. General locomotor activity was assessed using distance traveled, and the percent of time spent in the corners of the apparatus was used to determine anxiety-like behavior. Two mice were excluded from this behavioral test for being a statistical outlier via Grubb’s outlier test, resulting in a group size of 15–20/group.

### NORT

Two five-minute trials performed in the same open field apparatus within 1.5–2.5 hours of each other constituted the NORT. For the first trial, mice were placed in the box and allowed to explore two identical objects (rubber ducks). During the second trial, the right object was replaced with a novel object (saltshaker), and the animal was allowed to freely explore. The percentage of time the animal spent with the novel object relative to the total amount of time with both objects during the second trial was used to assess episodic memory. The intertrial interval was ~ 2 hours. Due to an interruption during testing for some cohorts, 36 mice were excluded because the intertrial interval was inconsistent with other cohorts. Further, one animal that spent less than 2 seconds exploring the objects was excluded from analysis (9–16/group).

### Barnes maze

Hippocampus-dependent spatial learning and memory were assessed using an eight-day variation of the Barnes Maze test. This protocol has been previously described in detail(14, 22). On the first day, 1 beaker trial was performed in which the mice were guided to the target hole using a clear beaker. Following the beaker trial on the first day and twice a day for the next three days, the mice performed learning trials to learn the target hole in which they were given three minutes to find the target hole, allowing them to escape upon finding the target. On the eighth day, the mice were subjected to a two-minute probe trial in which there was no escape after finding the target. During all trials, visual cues were displayed on the wall.

### Immunofluorescence

Mice were perfused with ice-cold 0.9% heparin saline. Brains were extracted and cut into right and left hemispheres. One half of the brain was post-fixed in 4% paraformaldehyde for 24 hours (side was determined by a flip of a coin), followed by submersion in 30% sucrose for at least 48 hours. Brains were then snap frozen in OCT (Thermo Fisher, 23–730-571) and stored at −80°C until sectioning. Brains were sectioned coronally at 35 microns on a Leica CM1950 cryostat into 7 series. A series sections were washed with PBS for 5 minutes 3 times. Slices were then transferred to a blocking and permeabilization buffer containing 0.3% Triton X-100 (Millipore, T9284) PBS (TPBS) solution with 10% donkey serum for one hour at room temperature. Following blocking, slices were incubated in primary antibody solution in 0.3% TPBS overnight in a cold room, containing 1:1000 goat Iba1 (Thermo Fisher, PA5–18039), 1:1000 rat CD68 (BIO-RAD, MCA1957), and 1:1000 rabbit GFAP (EMD Millipore, AB5804). Tissue was washed in PBS for 10 minutes 3 times before being incubated in secondary antibody solution in 0.3% TPBS for one hour at room temperature: 1:500 anti-goat 647 (Jackson Immuno Research, 705–605-147), 1:500 anti-rat Rhodamine Red-X (Jackson Immuno Research, 712–295-150), 1:500 anti-rabbit 488 (Jackson Immuno Research, 705–545-147), and 1:1000 DAPI (Thermo Fisher, D1306). Following treatment with secondary antibodies, tissue was washed twice in PBS for 10 minutes and once in PBS with 0.01% sodium azide (Krackeler, 45–71289-50G) for 15 minutes. Sections from another series were washed once with PBS for 10 minutes before being placed in a permeabilization buffer of 0.5% TPBS for 1 hour at room temperature. Then, slices were placed in a blocking buffer consisting of 0.5% TPBS and 4% donkey serum for 2 hours at room temperature. Following blocking, all sections were incubated in primary antibody solution, containing 1:500 rabbit Amyloid (Thermo Fisher, 71–5800) in 0.5% TPBS and 4% donkey serum, for 24 hours in a warm room. Slices were then washed for 10 minutes 3 times before incubation in secondary antibody solution for 2 hours in a warm room: 1:500 rabbit 488, 1:100 lectin 649 (Vector Laboratories, DL-1178–1), and 1:1000 DAPI in 0.5% TPBS with 4% donkey serum. After 2 hours, all sections were washed with PBS for 10 minutes twice and once with PBS with 0.1% sodium azide. All sections were mounted from anterior to posterior and cover slipped with 120uL of fluoromount-G. Slides were allowed to dry overnight before being stored at 4°C and imaged. Using the Axio Observer Fluorescent Microscope (Carl Zeiss Microscopy, Oberkochen, German), images of brain slices were obtained at 10x magnification using the same exposure times for each stain/labeling across all animals.

### Quantification of amyloid plaques and CAA

Using ImageJ (NIH), image brightness in each channel was adjusted to the same threshold for all animals. Plaques were quantified by measuring the percent area covered within each region of interest (ROI) by a blinded experimenter: the area of the retrosplenial cortex (rspCTX), stratum oriens of cornu ammonis 1 (CA1so), and ventral posterior thalamus (VP thal). In the same regions, CAA was assessed by measuring the percent area covered by pixels where amyloid and lectin colocalized. The value for each animal is representative of an average of two to three ROIs from sections containing the anterior, dorsal hippocampus. These regions are associated with memory, spatial learning, spatial processing, sensorimotor integration, and are known to be affected in AD and/or in the Tg-SwDI mouse model(23–26).

### Quantification of glia-related metrics

Similarly to previously described, ImageJ was utilized to set image brightness thresholds in each channel for all animals to assess microgliosis (Iba1), activated microglia (colocalization of Iba1 and CD68), and astrogliosis (GFAP). ROIs were drawn around the rspCTX, CA1so, polymorphic layer of the dentate gyrus (DGpo), and VP thal. Similarly, percent area covered averaged between two to three sections was utilized to assess the three modalities of neuroinflammation.

### Statistics

Statistical analyses were performed using Prism 10 (GraphPad Software, San Diego, CA, USA), with statistical significance set at *p* < 0.05. All data are shown as mean + SEM. Statistical outliers were assessed with Grubbs’ test, after which a 2-way ANOVA was performed with Fisher’s least significant difference in data segregated by sex, where strain (WT vs. Tg-SwDI) and diet (LFD vs. HFD) were the independent variables. To assess for sex differences, a separate 3-way ANOVA was performed without post-hoc analyses, such that strain, diet, and sex were the independent variables. For all metabolic data, a ROUT test was performed prior to analysis to assess for statistical outliers. Further, one-sample *t*-tests were performed to assess individual group performance relative to chance (50% in NORT, 15% in Barnes Maze). Correlations were assessed using Pearson’s correlation coefficient for the appropriate data sets.

## RESULTS

### HFD causes greater metabolic disturbances in AD and WT females compared to males

Previously, our lab has shown that HFD-induced metabolic syndrome is more severe in females in animal models of AD and vascular contributions to cognitive impairment and dementia (VCID)(14, 15, 21). In order to investigate these features in the Tg-SwDI model, WT and AD mice received either HFD or LFD from 3 months of age onward, and the GTT was used at 9 months to assess metabolic status (Fig. 1A). We demonstrate here that these sex differences are consistent in the Tg-SwDI AD model. For each sex, there was a main effect of diet on weight gain following 6 months of dietary intervention (p < 0.0001, Fig. 1B–C), on percent of visceral fat relative to total body weight (p < 0.001, Fig. 1D–E.), and on area under the blood glucose curve (AUC) during GTT (p < 0.0001, Fig. 1F–I). Further, there was a main effect of strain in both sexes on body weight at GTT (p < 0.01, Fig. 1B–C) and GTT AUC (p < 0.001, Fig. 1H–I), such that AD mice weighed more and had greater GTT AUC. Notably, prolonged exposure to a HFD induced a metabolic syndrome phenotype consistent with our previous findings, with elevated blood glucose levels after fasting (Fig. 1F–G at time = 0) and in response to a glucose challenge (Fig. 1H–I)(27, 28). The effects of strain and diet are sex dependent, demonstrated by post-hoc tests that show AD HFD females have greater metabolic impairment compared to WT HFD females (p < 0.01 for weight, % visceral fat, and GTT AUC) but AD LFD males have less body weight, % visceral fat, and GTT AUC compared to WT LFD males (p < 0.05 for all). Assessment of sex differences through a 3-way ANOVA (Additional File 1.) showed a significant effect of sex, diet, interaction between strain and sex, interaction between strain and diet, and interaction between sex and diet for all metabolic outcomes (p < 0.05). Altogether, these findings suggest AD females experience greater metabolic impairment in response to a HFD compared to WT females or WT or AD males.

### HFD worsens cognitive deficits in AD mice in a sex-specific manner

**Figure 2.**
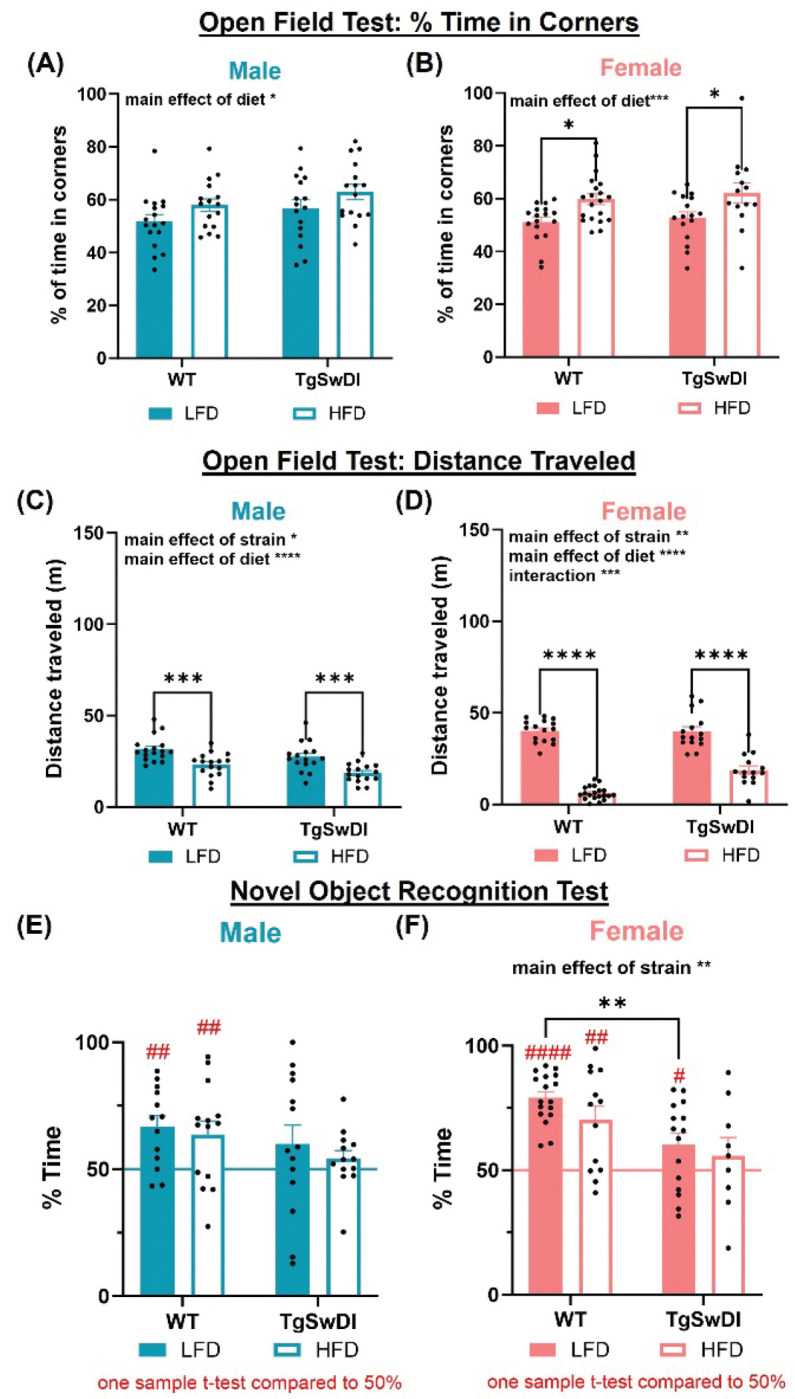
Diet and sex interact to affect anxiety-like behavior, general activity, and episodic memory. (A, B) Anxiety-like behavior was assessed through percent of time spent in the corners during the open field test and was increased in WT and Tg-SwDI animals receiving HFD across sexes. (C, D) Distance traveled during the open field test was used to assess general locomotor activity. HFD and AD pathology independently decreased the total distance traveled in both males (C) and females (D). (E, F) Episodic-like memory was assessed using the novel object recognition test (NORT). Percent of time spent with the novel object relative to total time spent with objects was calculated, such that performance significantly greater than 50% chance is indicative of intact memory. All AD males and HFD AD females did not perform significantly greater than chance. Pink and blue line = chance. * p<0.05, ** p<0.01, *** p<0.001, **** p<0.0001, # p<0.05 vs chance, ## p<0.01 vs chance, ### p<0.001 vs chance. Two-way ANOVA and one sample t-test (n=13–20/group).

Our previous research shows that diet differentially affects cognition in females in models of AD, VCID, and mixed dementia(15, 16, 21). Here, we show that these observations hold true in the Tg-SwDI model of AD and CAA. Percentage of time in corners during the open field test was used as an assessment of anxiety-like behavior. There was a significant main effect of diet in both males and females independently (p < 0.05, Fig. 2A–B), such that mice on HFD showed increased anxiety-like behavior. Post-hoc tests demonstrated significantly greater anxiety-like behavior in both WT and AD HFD females compared to their LFD-fed female controls (p < 0.05). Total distance traveled during the open field test was used as an assessment of general locomotor activity. In both males and females, there was a main effect of strain and diet (p < 0.05, Fig. 2C–D), with HFD-fed and AD mice independently traveling less. Post-hoc tests highlighted this effect, showing that all HFD-fed groups traveled smaller distances compared to their LFD-fed mice. Together, this demonstrates that HFD AD females specifically show increased anxiety-like behavior and HFD-fed mice traveled less.

Recognition memory was assessed in the novel object recognition test (NORT). Preference for the novel object in the NORT is measured as the recognition index: percent of time spent with the novel object relative to total time spent with objects. Performance not greater than 50% chance indicates impairment in recognition memory. Assessed individually within groups, HFD-fed AD females and all AD males did not perform significantly greater than chance (p > 0.05, Fig. 2E–F), indicating impaired recognition memory. Comparisons between groups demonstrated a main effect of strain in the females (p < 0.01, Fig. 2F), such that AD females spent significantly less time exploring the novel object, indicating more severely impaired memory. Together, this suggests that HFD-fed AD mice show impairment in recognition memory regardless of sex.

**Figure 3.**
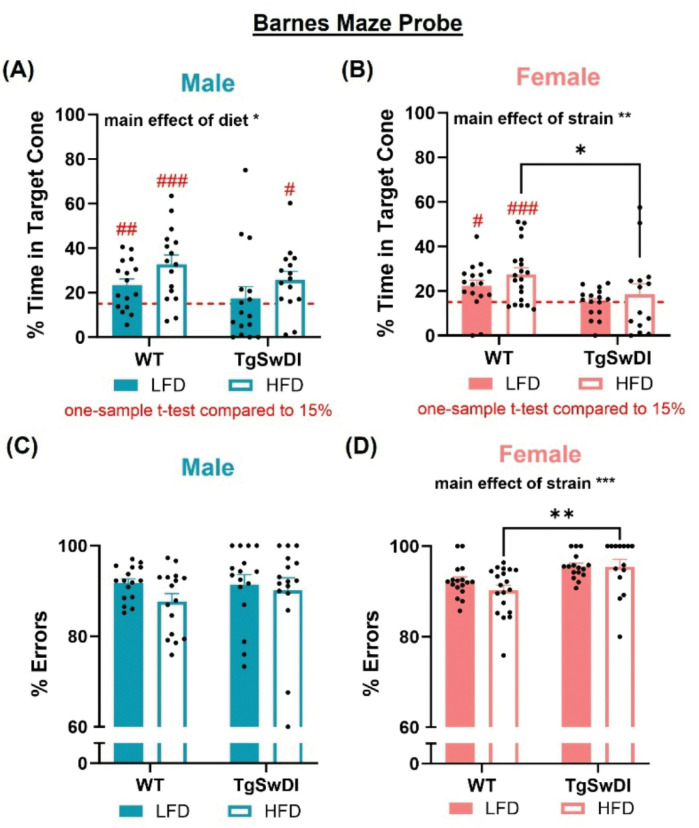
HFD impairs spatial memory in female Tg-SwDI animals. Spatial learning and memory were assessed using the Barnes maze. Spatial memory was assessed through the probe trial (A-D) through percent of time in the cone adjacent to the target, such that performance above 15% chance was indicative of greater learning, (A, B) and percent of entries in the incorrect holes relative to the correct hole (C, D). TgSwDI females spent less time in the target cone (F) and had greater percentage of erroneous entries (H) during the probe trial compared to WT females. Red line = chance. * p<0.05, ** p<0.01, *** p<0.001, **** p<0.0001, # p<0.05 vs chance, ## p<0.01 vs chance, ### p<0.001 vs chance. Two-way ANOVA and one sample t-test (n=14–20/group).

The Barnes maze test was used to assess spatial learning and memory via the hidden trials and probe trial, respectively. The percentage of time spent in the portion of the maze between the center and holes directly adjacent to the target hole (target cone) and percentage of incorrect hole entries were used to assess performance. In the probe trial, to assess spatial memory, percent of time in the target cone was assessed in each group independently to compare performance to chance, which is 15% of time spent in the target cone. All AD females and HFD-fed AD males did not perform significantly different to chance (p > 0.05, Fig. 3A–B), indicating impairment in spatial memory. Comparisons between groups revealed a main effect of diet in the males (p < 0.05, Fig. 3A) and a main effect of strain in the females (p < 0.01, Fig. 3A), such that HFD-fed males spent more time in the target cone and AD females spent less time in the target cone. Further, there was a main effect of strain on percent errors in the probe trial in females (p < 0.01, Fig. 3D), such that AD females made greater errors. Post-hoc comparisons showed that AD HFD-fed females made significantly more errors than WT HFD-fed females (p < 0.01). Interestingly, during spatial learning, there was a significant effect of diet in the males and a significant effect of strain in the females (p < 0.05, Additional File 2C-D), such that HFD and WT strain independently resulted in less errors during learning in males and females, respectively. Taken together, these findings demonstrate that AD mice receiving HFD show impairment in spatial memory and that these observations are particularly strong in HFD-fed AD females.

### AD-induced neuroinflammation is tempered by HFD in females

**Figure 4.**
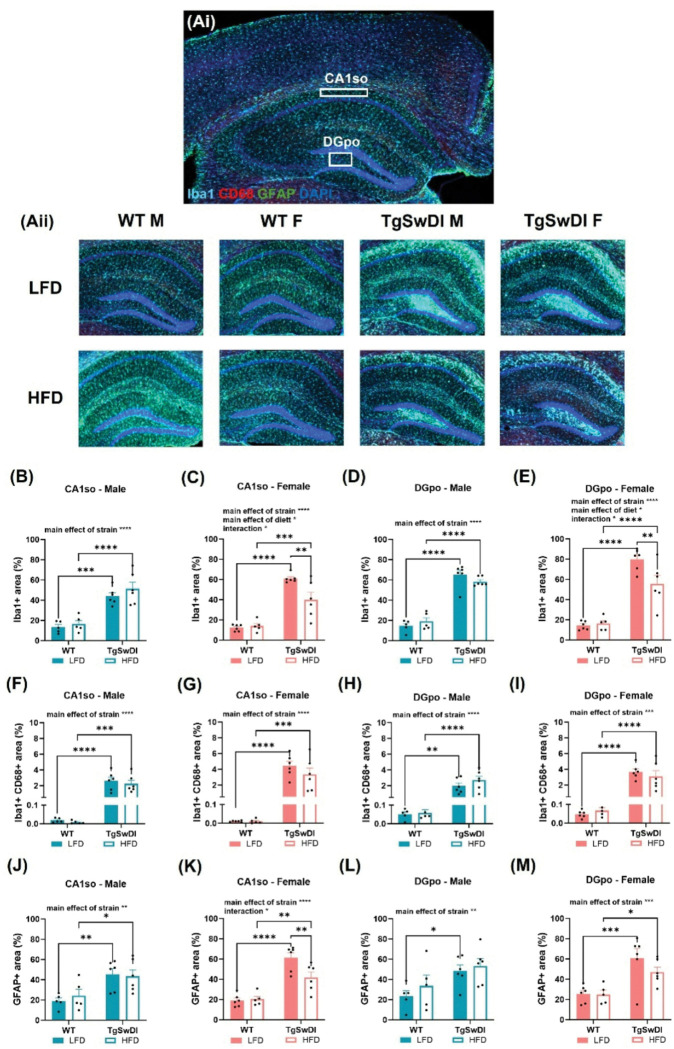
AD, diet, and sex interact to affect microgliosis, activated microglia, and astrogliosis in the hippocampus. (Ai) Hippocampal regions of interest examined included the stratum oriens layer of the cornu ammonis 1 (CA1so) and the polymorphic layer of the dentate gyrus (DGpo) in anterior coronal sections. (Aii) Representative images of Iba1, CD68, GFAP, and DAPI fluorescence in the hippocampus are shown. (B-E) Microgliosis was assessed through Iba1 immunofluorescence by percent of area covered in the region of interest. (F-I) Activated microglia was assessed by colocalizing Iba1 and CD68 immunofluorescence and quantifying the percent of colocalized fluorescence in the region of interest. (J-M) Astrogliosis was examined through GFAP immunofluorescence via percent of area covered in the region of interest. * p<0.05, ** p<0.01, *** p<0.001, **** p<0.0001. Two-way ANOVA (n=5–6/group).

Previously, our lab has investigated neuroinflammation in other models of AD with and without comorbid metabolic dysfunction(15, 16, 21, 22). Here, we sought to expand upon these observations in the Tg-SwDI model that contains CAA pathology. Activated microglia was assessed as percent of the region of interest covered by cells immunolabeled with both Iba1 and CD68, due to CD68 being an indication of active phagocytosis. Similarly, microgliosis and astrogliosis were quantified by percent of the region of interest covered by Iba1-immunolabeled or GFAP-immunolabeled cells. Given previous findings that suggest hippocampal neuroinflammation is involved in AD pathology, we first examined these measures of inflammation in the stratum oriens layer of cornu ammonis 1 (CA1so) and the polymorphic layer of the dentate gyrus (DGpo, Fig. 4A)(29). In both regions and in males and females, there was a main effect of strain on microgliosis (p < 0.0001, Fig. 4B–E), such that AD males and females showed increased area of microglia regardless of diet. Further, in both regions AD females receiving HFD showed less microgliosis compared to AD females receiving LFD (p < 0.01, Fig. 4C. and Fig. 4E). Interestingly, there was similarly a main effect of strain on activated microglia and astrogliosis across sexes (p < 0.01, Fig. 4F–M, resulting in increased area covered by activated microglia and astroglia in hippocampal tissue from AD mice. In CA1so, post-hoc tests reveal that HFD-fed AD females show significantly less astrogliosis compared to LFD-fed AD females (p < 0.01, Fig. 4K). These trends were consistent in the ventral posterior thalamus (VP thal), which was an area of interest due to its implication in CAA pathology in this model(19, 26, 30). Specifically, post-hoc tests showed that microgliosis and activated microglia were significantly decreased in the VP thal of HFD-fed AD females compared to control AD females (p < 0.05, Additional File 3C and Additional File 3E). Further, a 3-way ANOVA was performed on each metric of inflammation to assess for sex differences. In CA1so, there was a main effect of sex and an interaction between strain and sex on activated microglia, as well as an interaction between sex and diet and interaction between strain, sex, and diet on microgliosis (p < 0.05, Additional File 1.). This resulted in greater overall neuroinflammation in female AD mice compared to male AD mice. Overall, these results suggest that the Tg-SwDI mice have more hippocampal neuroinflammation than WT mice and that HFD decreases neuroinflammation in some regions in AD females.

### HFD exacerbates pathology in the thalamus of AD females

**Figure 5.**
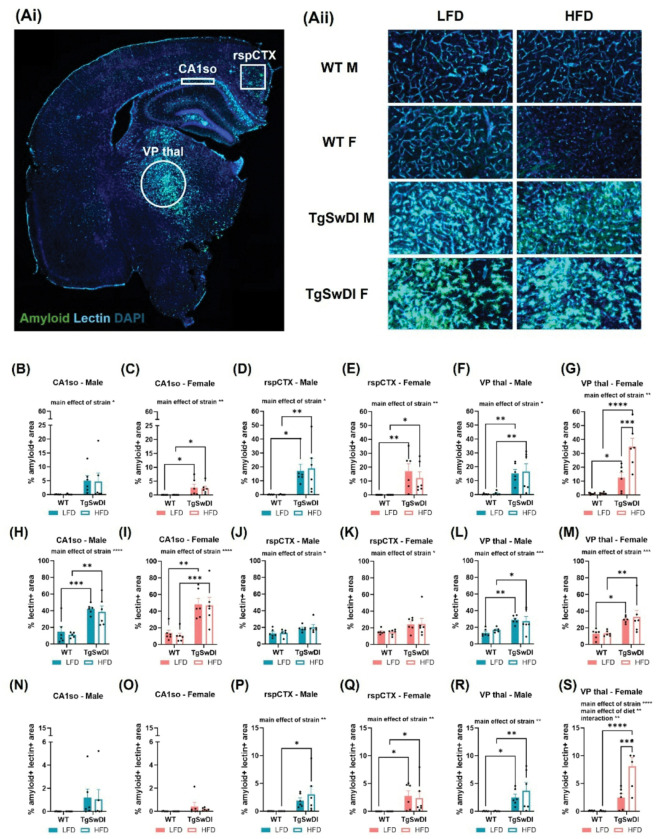
Sex and diet interact to affect CAA pathology and blood vessel density. (Ai) Regions of interest in the hippocampus (CA1so), cortex (retrosplenial cortex, rspCTX), and thalamus (ventral posterior thalamus, VP thal) are shown in an anterior coronal section. (Aii) Representative images of amyloid β, lectin, and DAPI fluorescence are shown in the VP thal. (B-G) AD pathology was assessed via percent of area of interest containing amyloid β immunofluorescence. (H-M) Blood vessel density was assessed through lectin fluorescence by percent of area covered in the region of interest. (N-S) CAA pathology was measured via colocalization of amyloid β and lectin fluorescence and assessed by the percent of the region of interest covered by the colocalized fluorescence. * p<0.05, ** p<0.01, *** p<0.001, **** p<0.0001. Two-way ANOVA (n=5–6/group).

While we have previously examined the effect of HFD on neuropathology in other AD and dementia models, this study is the first to examine how comorbid metabolic dysfunction affects CAA pathology in the Tg-SwDI model(15, 21, 22). Extensive research demonstrates the hippocampus and cortex are heavily burdened by amyloid pathology and additional findings show the cortex and thalamus also contain significant CAA in the Tg-SwDI model(29, 30). As such, we examined pathology in the CA1so, retrosplenial cortex (rspCTX), and VP thal. We measured CAA by quantifying the area of a region of interest containing amyloid plaques colocalized with blood vessels, using lectin staining. Similarly, we also assessed amyloid deposition and vessel density using the percent of the region containing amyloid-tagged plaques or lectin staining. Unsurprisingly, across all regions and in both males and females, there was a main effect of strain on amyloid deposition (p < 0.05, Fig. 5.B–G), such that tissue from WT mice contained no amyloid. Interestingly, post-hoc tests revealed a significant increase in amyloid in the VP thal of HFD-fed AD females compared to LFD-fed AD females (p < 0.001, Fig. 5G). In all regions, there was also a main effect of strain on blood vessel density (p < 0.05, Fig. 5H–M), such that AD males and females had increased vascular density compared to WT controls. In the rspCTX and VP thal, there was a main effect of strain in both males and females (p < 0.05, Fig. 5P–S.), indicating CAA is only observed in tissue from AD mice. Further, post-hoc tests show that AD females receiving HFD have greater CAA in the VP thal compared to AD females receiving LFD. Taken together, these findings show that exposure to HFD increases amyloid deposition and CAA in the thalamus in AD females and AD increases vascularity across several brain regions in both sexes.

### Weight correlates with CAA pathology in the thalamus of AD females

**Figure 6.**
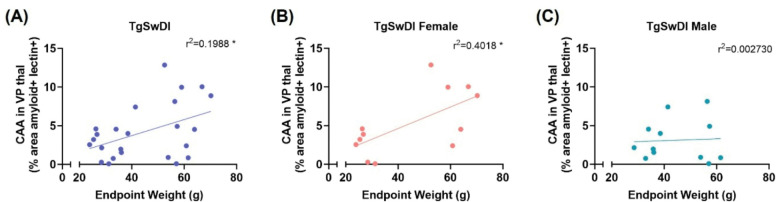
CAA pathology in the thalamus correlates with endpoint weight in AD females. The percent area of VP thal covered by colocalized fluorescence of amyloid β and lectin were correlated with endpoint weight in all AD animals (A), AD females (B), and AD males (C). When analyzing all AD animals together, there was a significant correlation between CAA and endpoint weight (A), and when sex was considered it was revealed that this positive correlation was consistent in AD females (B) but not males (C). * p<0.05. Linear regression with Pearson’s correlation coefficient (n=43)

To assess how HFD-induced obesity related to thalamic CAA pathology across sexes, we performed correlations between weight at the end of study and CAA pathology first in all AD mice and then separately in AD males and females. When data were pooled across sexes, there was a significant positive correlation between CAA pathology and endpoint weight (r^2^ = 0.1988, p < 0.05, Fig. 6A). However, when assessed separately, only AD females demonstrated significant positive correlations between weight and CAA pathology (r^2^ = 0.4018, p < 0.05, Fig. 6B). These results suggest that HFD exacerbates vascular dementia pathology, specifically in females.

## DISCUSSION

This study sought to better understand the effect of endocrine risk factors, such as sex and metabolic disease, on AD and VCID by examining metabolism, cognition, and neuropathology. Evidence shows that women are more likely to develop AD, likely due to underlying differences in metabolism, phagocytosis, and immune response that change during aging(31). Further, metabolic risk factors such as metabolic syndrome, prediabetes, and obesity are known to increase the risk of developing VCID, with these comorbidities confounding the risk for women(2, 3, 32). How exactly sex and metabolic syndrome interact to affect cognition and pathology is unknown in the Tg-SwDI model of AD and VCID (cerebral amyloid angiopathy). We used chronic HFD administration to model obesity and metabolic syndrome. While all animals developed impaired glucose tolerance, female mice experienced greater metabolic disturbances. Further, these sex differences were exacerbated in AD mice. Similarly, metabolic syndrome resulted in greater cognitive impairments in AD females compared to males. While HFD increased anxiety-like behavior and episodic memory across sexes in AD animals, females also experienced disturbances in spatial memory. Interestingly, HFD decreased hippocampal microgliosis and astrogliosis but increased thalamic amyloid plaques and CAA pathology in AD females, with little effect on neuropathology in the males. Together, these results suggest that females with AD are more vulnerable to metabolic, cognitive, and pathologic effects of diet-induced metabolic syndrome. This, coupled with previous findings, support the idea that metabolic disease may differentially increase the risk of developing dementia and alter the disease process in women compared to men(2).

In this study, we found that AD females had more severe metabolic impairment in response to chronic HFD administration. Specifically, HFD resulted in greater weight gain, visceral fat accumulation, and glucose intolerance in AD females compared to males. This is consistent with previous findings in our lab and others in other models of AD and VCID(15, 21, 27, 33, 34). Previously, we have shown that a similar diet regimen resulted in changes in the periphery in AD males and females: hepatic fibrosis, steatosis, and increases in circulating leptin. However, in the hypothalamus, levels of GFAP and interleukin-1β were greater in females and associated with their increased weight gain in response to a HFD, suggesting neuroinflammation in this region may contribute to metabolic sex differences(21). These observations are consistent with trends in AD patients, where women are thought to be more susceptible to metabolic disease and diabetes(3). Interestingly, we also found sex differences in our LFD-fed, control AD males and females, such that the AD males weighed less, had less visceral fat, and had greater glucose tolerance than WT males. Conversely, LFD-fed AD females weighed more and had worse glucose tolerance compared to WT females. This is also consistent with human data, which shows that in mid-adulthood underweight men and overweight females have greater risk of developing AD(35). Additionally, previous research has demonstrated that mutant amyloid precursor protein (APP) differentially alters lipid metabolism in the periphery in control vs obesogenic settings in AD and CAA models, suggesting a mechanism for these metabolic differences(36). Further, recent findings demonstrated that glucagon-like peptide-1 therapy conjugated with estradiol can improve HFD-induced metabolic, cognitive, and pathologic deficits via distinct sex-specific mechanisms(37). Together, these findings suggest that underlying sex differences in metabolism may be exacerbated by AD and vascular dementia, necessitating the need to better understand how these differences may be modulated to target sex-specific effects of disease.

We also demonstrated diet-driven sex differences in cognition in this model. Specifically, we showed that HFD increased anxiety-like behavior, decreased exploration, and impaired recognition memory in males and females but that spatial memory was impaired only in AD females. These cognitive sex differences are similar to what we have shown previously in other models of AD and VCID(15, 16, 21, 22). Interestingly, in both sexes, HFD was sufficient to induce increased anxiety-like behavior and decreased mobility, and these trends were exacerbated in AD animals. This is consistent with previous findings that model obesity-driven changes in affect(38). However, in assessments of memory, AD and/or HFD were required to induce impairments. For recognition memory, all AD males showed impairment, but in females only AD HFD animals were impaired. Although, AD females on control diet did demonstrate worse recognition memory compared to WT controls. Interestingly, men and women are known to have differing strengths in performance in metrics of memory(39). One study found that women with family history of AD consistently performed better on episodic memory tasks compared to men with a positive family history(40). In assessments of spatial memory, HFD AD females experienced significant impairment, whereas HFD AD males did not perform differently than control AD males. Recent findings in another model of AD showed similar pronounced spatial deficits in AD females compared to males(41). Our findings supplement these observations of sex-specific cognitive deficits by demonstrating that many of these features are exacerbated by HFD. Additionally, these data suggest that the combined effect of metabolic disease and AD often result in cognitive impairments more severe than each individual insult.

When assessing for neuroinflammation, we found that microgliosis, activated microglia, and astrogliosis were all consistently elevated in AD animals. Interestingly, HFD appeared to temper neuroinflammation in AD females, while HFD had little effect on AD males. Previous work in our lab modeling metabolic disease in AD models has shown differences in neuroinflammatory response between males and females(15, 21). This and ongoing aging research support the notion that sex differences may contribute to underlying differences in metabolism, immune reactivity, and autophagy(31, 42, 43). However, our results suggest that both diet-induced metabolic disease and VCID together, specifically in females, may impair immune responsivity in the brain. Recent studies have demonstrated a similar phenomenon of immune exhaustion in AD, showing that some T cells and microglia become exhausted by pathology and that these conditions result in worsening cognitive impairment(44–46). Additionally, some research suggests that immune exhaustion in AD and other diseases is modulated by diet-induced obesity and intrinsic, molecular sex differences(45, 47). Further, imaging studies in patients suggest that prediabetic women experience cerebral hypometabolism to a greater extent than men(32). It is possible that this holds true in mice as well, suggesting that metabolic disease may impair metabolism in the brain, which could affect mobilization of neuroinflammatory cells. While microglia specifically have been heavily implicated in AD pathology, AD models that lack microglia are shown to have greater amyloid and CAA pathology and early lethality(48). These findings, coupled with our observations of decreased microgliosis and astrogliosis in AD HFD females, suggest that metabolic disease may worsen pathology by impairing appropriate immunoreactivity in a sex-specific manner.

Our assessment of pathology demonstrated that HFD exacerbates total brain amyloid beta accumulation and CAA pathology in females but not males. Further we showed that animal weight at the end of study correlated with thalamic CAA pathology again in females but not males. Previous research in this Tg-SwDI model has shown prominent plaque accumulation in the cortical parenchyma, as well as in the vasculature in the thalamus(30). Additionally, accumulation of activated microglia, reactive astrocytes, and complement proteins were found adjacent to the CAA pathology(19, 30). These findings suggest neuroinflammation and vasculature pathology coexist and exacerbate each other. Recent work in both clinical and animal models has shown that vascular amyloid interacts with monocytes to promote complement-mediated blood-brain barrier injury(49). This indicates that inflammatory components may actually directly contribute to the spread of pathology, specifically within the vasculature. As mentioned above, in AD models that lack microglia, CAA pathology is exacerbated, suggesting that existence of some microglia is required to mitigate pathology(48). This study is the first to demonstrate that diet-induced metabolic syndrome may minimize neuroinflammation while exacerbating CAA pathology in females, further complicating the relationship between local inflammation and AD pathology. Additionally, we found that our AD mice, regardless of diet, had consistently greater blood vessel density in our areas of interest (hippocampus, cortex, and thalamus) compared to WT animals. Interestingly, the most potent vascular permeability factor, vascular endothelial growth factor (VEGF), has previously been implicated in AD and CAA pathology, such that selectively inhibiting three of its receptors resulted in decreased amyloid deposition in vessels and decreased glial reactivity(50). Together with our results, this suggests that HFD may enhance pathology-related blood vessel growth in a sex-specific manner. Altogether, our results show that diet-induced metabolic disease worsens disease pathology by both increasing amyloid and blood vessel density, ultimately increasing the amount of vascular amyloid.

## CONCLUSION

To our knowledge, this is the first study performed in the Tg-SwDI model to investigate interactions between diet-induced metabolic disease and sex on AD and VCID. We demonstrated that across metabolic, cognitive, and pathologic findings, AD females were consistently more vulnerable to HFD-induced deficits. Further, we showed for the first time in this model that diet aggravates amyloid and CAA pathology in multiple ways, while attenuating neuroinflammation, in females. Our findings add pertinent detail about metabolic and sex risk factors in VCID that is similar to previous findings in AD. This work supports the importance of understanding how women may be at higher risk of metabolic disease and comorbid dementia. Future studies further elucidating pathophysiology in this unique array of comorbidities is necessary to identify ideal therapeutic and preventative tools for dementia.

## Supplementary Material

Supplementary Files

This is a list of supplementary files associated with this preprint. Click to download.

• AdditionalFile1.xlsx

• AdditionalFile3.jpg

• AdditionalFile2.jpg

## Data Availability

The datasets used and analyzed during this study are available from the corresponding author upon request.

## Abbreviations

AD: Alzheimer’s disease
CAA: cerebral amyloid angiopathy
LFD: low fat diet
HFD: high fat diet
WT: wild type
Tg-SwDI: C57BL/6-Tg(Thy1-APPSwDutIowa)BWevn/Mmjax
GTT: glucose tolerance test
Triton X-100 PBS: novel object recognition test (NORT)
TPBS: region of interest
ROI: retrosplenial cortex (rspCTX)
CA1so: stratum oriens of cornu ammonis 1
VP thal: ventral posterior thalamus
DGpo: dentate gyrus
AUC: area under the curve
